# Mini-access ascending aorto-bifemoral bypassing with tricuspid annuloplasty: A novel approach for mid-aortic syndrome combined with valvular disease

**DOI:** 10.1016/j.xjtc.2025.02.002

**Published:** 2025-02-15

**Authors:** Seohee Joo, Byeong A. Yoo, Kitae Kim, Joon Bum Kim

**Affiliations:** Department of Thoracic and Cardiovascular Surgery, Asan Medical Center, University of Ulsan College of Medicine, Seoul, Republic of Korea

**Figure:**
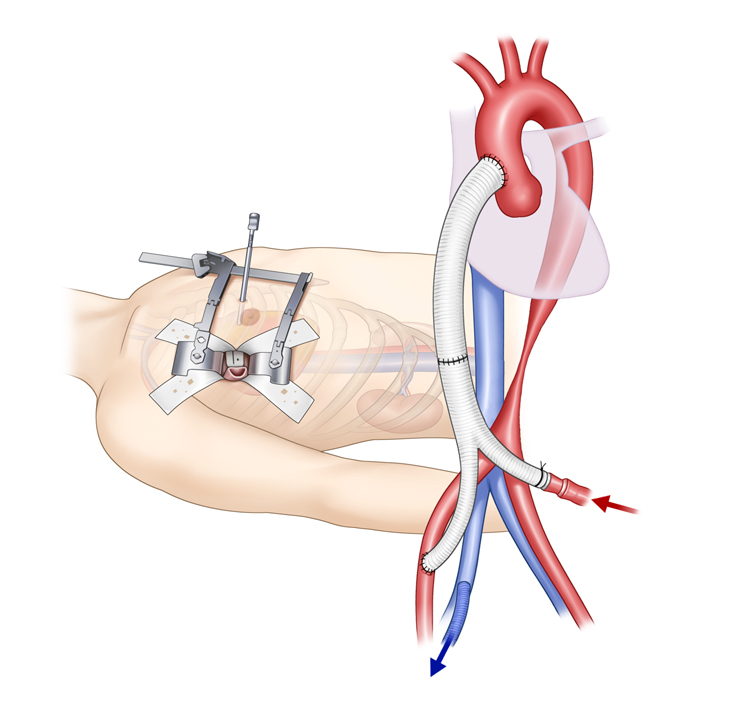
Mini-access ascending aorto-bifemoral bypass with tricuspid annuloplasty

**Central Message**

This novel approach involving mini-access ascending aorto-bifemoral bypass with tricuspid annuloplasty is safe and effective.

Extra-anatomic bypass surgery using the ascending aorta as the inflow source is a highly effective treatment for aorto-iliac steno-occlusive diseases, such as mid-aortic syndrome.^1^^,^^2^ However, a central bypass usually requires extensive incisions involving both a sternotomy and laparotomy, and thus pose significant challenges in postoperative pain management and postoperative recovery.^3^^,^^4^ Especially in an era of advanced minimally invasive and transcatheter techniques, large incisions may deter patients from opting for surgical because of cosmetic concerns.^5^ Furthermore, when treating mid-aortic syndrome combined with valvular heart disease, management of cardiopulmonary bypass poses an additional challenge, because femoral cannulation is likely to hamper perfusion to the upper body. Here we present a novel approach as a minimally invasive alternative: a mini-access ascending aorto-bifemoral bypass combined with tricuspid valve repair, performed using laparoscopic and thoracoscopic techniques.

## Case Presentation

**Figure 1:**
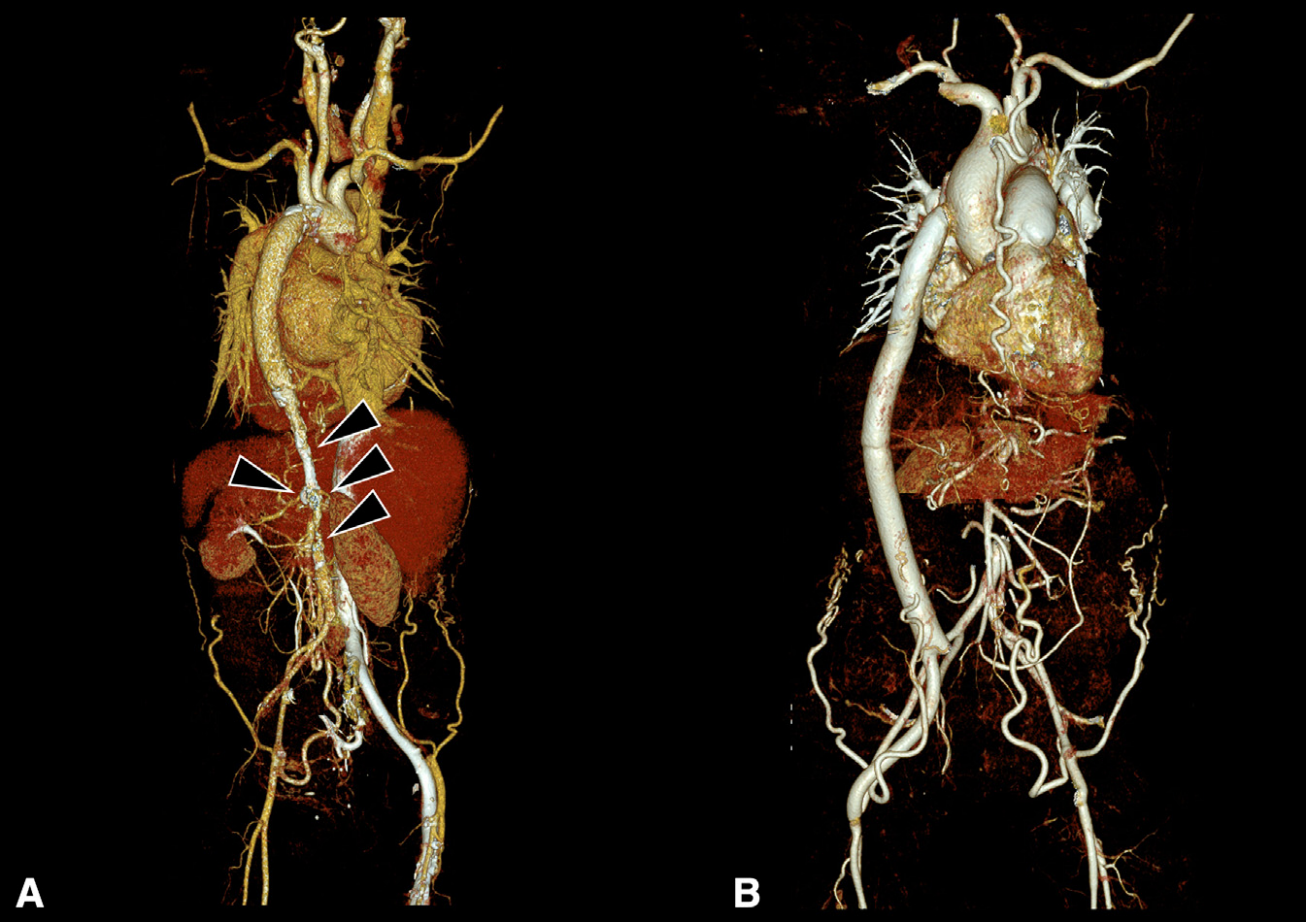
Three-dimensional computed tomography (*CT*) images. A, Posterior view of preoperative CT scan showing multifocal luminal narrowing of the descending thoracic aorta, abdominal aorta, and both renal arteries. B, Anterior view of postoperative CT scan showing a patent bypass graft.

A 58-year-old female with mid-aortic syndrome due to Takayasu arteritis presented with severe dyspnea with peripheral edema. Chest radiography revealed marked cardiomegaly. Laboratory findings showed a reduced glomerular filtration rate of 32 mL/kg/min/1.73 m^2^. Echocardiography demonstrated severe left ventricular (LV) dysfunction, with an LV ejection fraction of 26% and moderate functional mitral regurgitation, likely due to escalated afterload to the LV. Severe tricuspid regurgitation (TR) also was observed, accompanied by severe resting pulmonary hypertension (peak TR velocity, 4.9 m/s). The ankle-brachial index was measured at 0.45 on the left side and 0.46 on the right side. A computed tomography (CT) scan revealed multifocal luminal narrowing of the descending thoracic and abdominal aorta as well as both renal arteries, consistent with chronic Takayasu arteritis (Figure 1, *A*). The need for Ethical Committee approval was waived owing to the nature of the study, and informed consent for publication was obtained from the patient.

## Surgical Technique

An aorto-bifemoral bypass and tricuspid valvuloplasty were performed closely following the surgical techniques described in previous reports. A long bypass conduit was preconstructed from commercially available polyester grafts by suturing a straight graft (Hemashield Platinum Woven Double Velour, straight graft, 18 mm; Getinge) to a bifurcated graft (Hemashield Platinum Woven Double Velour, bifurcated graft, 18 mm × 9 mm; Getinge) with the same diameter to form a sufficiently long Y-graft. Graft sizes were determined based on the dimensions of the ascending aorta and femoral artery from preoperative CT scans. A right anterolateral mini-thoracotomy in the fourth intercostal space and a 10-mm thoracoscopic port were created. After a pericardiotomy, the ascending aorta was dissected and snared under thoracoscopic guidance.

**Figure 2:**
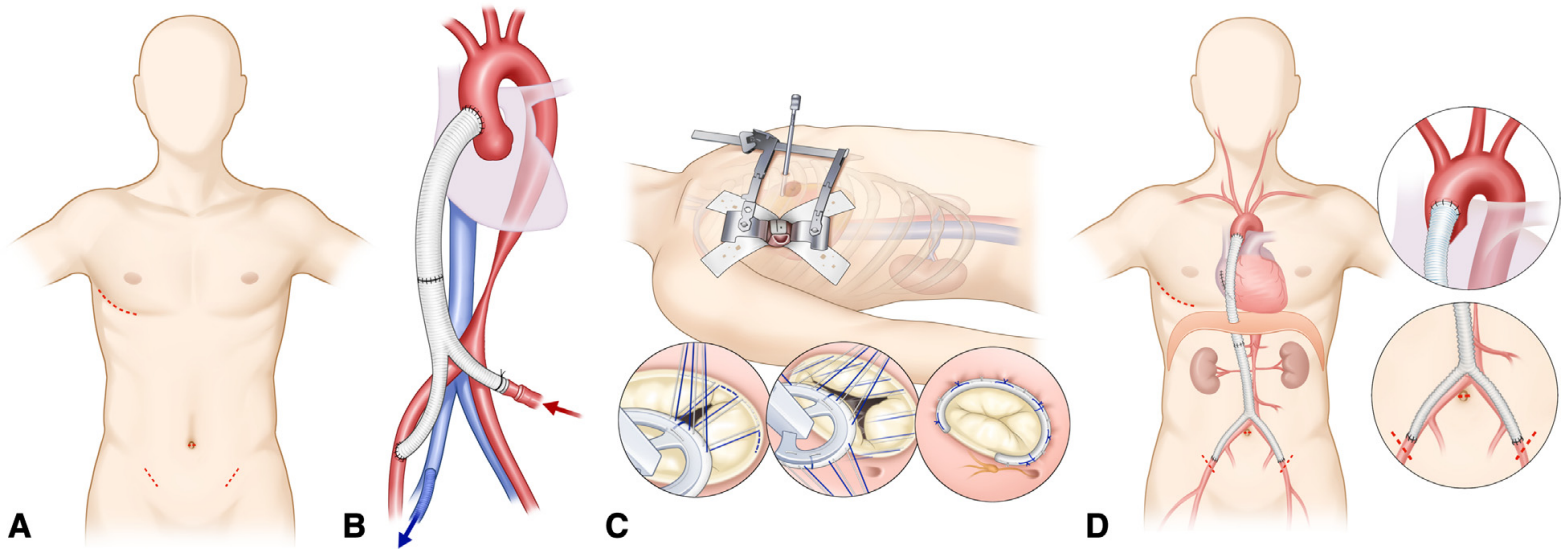
Schematic drawings of the mini-access ascending aorto-bifemoral bypass with tricuspid annuloplasty. A, Limited skin incisions: a right anterolateral mini-thoracotomy and minimal incisions in the umbilicus and both groins. B, Cardiopulmonary bypass inflow through the bifurcated graft limb and outflow via the right femoral vein. C, Beating heart tricuspid annuloplasty via a right anterolateral mini-thoracotomy D, Final graft configuration.

An additional laparoscopic port was formed via a 10-mm trocar incision at the umbilicus, and both femoral arteries were exposed via 2-cm incisions in both inguinal areas (Figure 2, *A*). Under laparoscopic guidance, the diaphragm was punctured from the chest cavity. The bifurcated graft limbs were passed laparoscopically from the umbilical port to the femoral site, and the straight graft was routed from the peritoneal to the thoracic cavity via the punctured diaphragm. Graft configuration was verified laparoscopically to ensure no torsion or kinking. The course of the graft lying on the greater omentum also was validated by laparoscopic visualization.

The ascending aorta was partially clamped and proximal anastomosis to the ascending aorta was performed with a 4-0 polypropylene running suture and pledgeted reinforcements, whereas distal anastomosis to the right femoral artery used a 5-0 polypropylene running suture (Video 1). Cardiopulmonary bypass (CPB) was established with inflow through the remaining bifurcated graft limb and outflow via the right femoral vein (Figure 2, *B*). A multichannel femoral drain-catheter was used for the outflow cannula and placed along the inferior vena cava and superior vena cava with the tip positioned at the mid-superior vena cava to facilitate effective suction drainage of venous return. Beating-heart tricuspid annuloplasty was performed using a 26-mm MC3 ring (Edwards Lifescience) under thoracoscopic guidance (Figure 2, *C*). After weaning off CPB, the remaining graft limb was anastomosed to the left femoral artery (Figure 2, *D*).

The patient's postoperative course was uneventful. She was transferred to the general ward on postoperative day 1 and discharged on postoperative day 11 without complications. Postoperative echocardiography showed improved LV function (LV ejection fraction 47%), trivial mitral regurgitation, mild TR, and relieved pulmonary artery pressure. Postoperative CT (Figure 1, *B*) confirmed a patent bypass conduit and an optimal graft configuration. Postoperative ankle-brachial index improved to 0.95 on the left side and 0.96 on the right side. Renal function recovered to a glomerular filtration rate of 53 mL/kg/min/1.73 m^2^.

## Discussion

Mid-aortic syndrome is a rare condition characterized by narrowing of the distal thoracic and/or abdominal aorta, causing hypertension proximal to the lesion and hypotension distal to the lesion.^1^ Severe arterial hypertension occurs in approximately 90% of affected individuals, and congestive heart failure from increased afterload is reported in 2.5% to 26.7% of cases.^E1^^,^^E2^ Renal involvement is common, with 69% to 80% of patients exhibiting renal artery stenosis and 20.5% developing renal impairment from renal ischemia and hypertension-related damage.^E3^

Surgical approaches for mid-aortic syndrome include patch aortoplasty, aortic replacement, and extra-anatomic bypass grafting, with surgery offering longer freedom from reintervention and fewer complications compared to percutaneous interventions.^E4^ Extra-anatomic bypass grafting is considered safe and effective.^E1^ Central bypasses typically require extensive incisions, whereas peripheral vessel bypasses are associated with poor long-term patency. To mitigate the challenges associated with large incisions in central bypasses, minimal invasive central bypass methods have been adopted.

Mini-access ascending aorto-bifemoral bypass has several demonstrated advantages, including a lower rate of transfusion, reduced wound problems, less postoperative pain, and rapid physical recovery.^E5^ Although this approach offers significant benefits, careful patient selection is important, particularly in cases with severe pleural or peritoneal adhesions, a small ascending aorta unsuitable for safe side-clamping, or a severely atherosclerotic aorta with a high risk of atheroembolism, limiting direct manipulation.

Simultaneous surgical treatment of mid-aortic syndrome and valvular disease demands careful systemic perfusion strategies. Arterial cannulation is crucial, given that right axillary or ascending aorta cannulation ensures cerebral perfusion but risks visceral organ malperfusion, while femoral artery cannulation maintains visceral perfusion but may interrupt cerebral blood flow. In the present case, double cannulation using the bifurcated graft limb as the inflow cannula provided continuous blood flow both proximal and distal to the stenosis, maintaining sufficient cerebral and visceral organ perfusion. However, this approach is not suitable for patients with mid-aortic syndrome and left-sided valvular disease, as these cases require ascending aortic cross-clamping. Once the ascending aorto-graft anastomosis is established, no space remains for aortic clamping. An alternative strategy involves (1) clamping the distal ascending aorta, (2) completing the aortic or mitral procedure, and (3) performing the proximal bypass anastomosis. A limitation of this approach is the need for an additional arterial inflow site, most likely the right axillary artery, because femoral cannulation alone does not ensure adequate perfusion to the upper body. Although clinical evidence is currently lacking, this approach remains a potential surgical option for select patients with mid-aortic syndrome and left-sided valvular disease.

Given the underlying pathophysiology, coexistence of valvular disease and mid-aortic syndrome is likely to occur, making this technique a significant point of reference. This approach effectively reversed hypertrophic cardiac remodeling in our patient and resulted in improved renal function and relief of ischemia in the lower limbs.

## Conclusions

This case demonstrates a novel, safe, and effective technique for revascularization and valve repair in mid-aortic syndrome with concomitant valvular pathology, supporting surgical treatment as a critical strategy for achieving optimal outcomes.

## Conflict of Interest Statement

The authors reported no conflicts of interest.

The *Journal* policy requires editors and reviewers to disclose conflicts of interest and to decline handling or reviewing manuscripts for which they may have a conflict of interest. The editors and reviewers of this article have no conflicts of interest.

## Supplementary Data

Video 1 Comprehensive visualization of mini-access ascending aorto-bifemoral bypass with tricuspid annuloplasty in this patient with mid-aortic syndrome and severe tricuspid regurgitation. Video available at: https://www.jtcvs.org/article/S2666-2507(25)00068-9/fulltext.
