# Supplementary figures and images for: Mini-access ascending aorto-bifemoral bypassing with tricuspid annuloplasty: A novel approach for mid-aortic syndrome combined with valvular disease
Source: JTCVS Tech. 2025 Feb 15;30:18–22. doi: 10.1016/j.xjtc.2025.02.002 (PMC11998586; doi:10.1016/j.xjtc.2025.02.002)

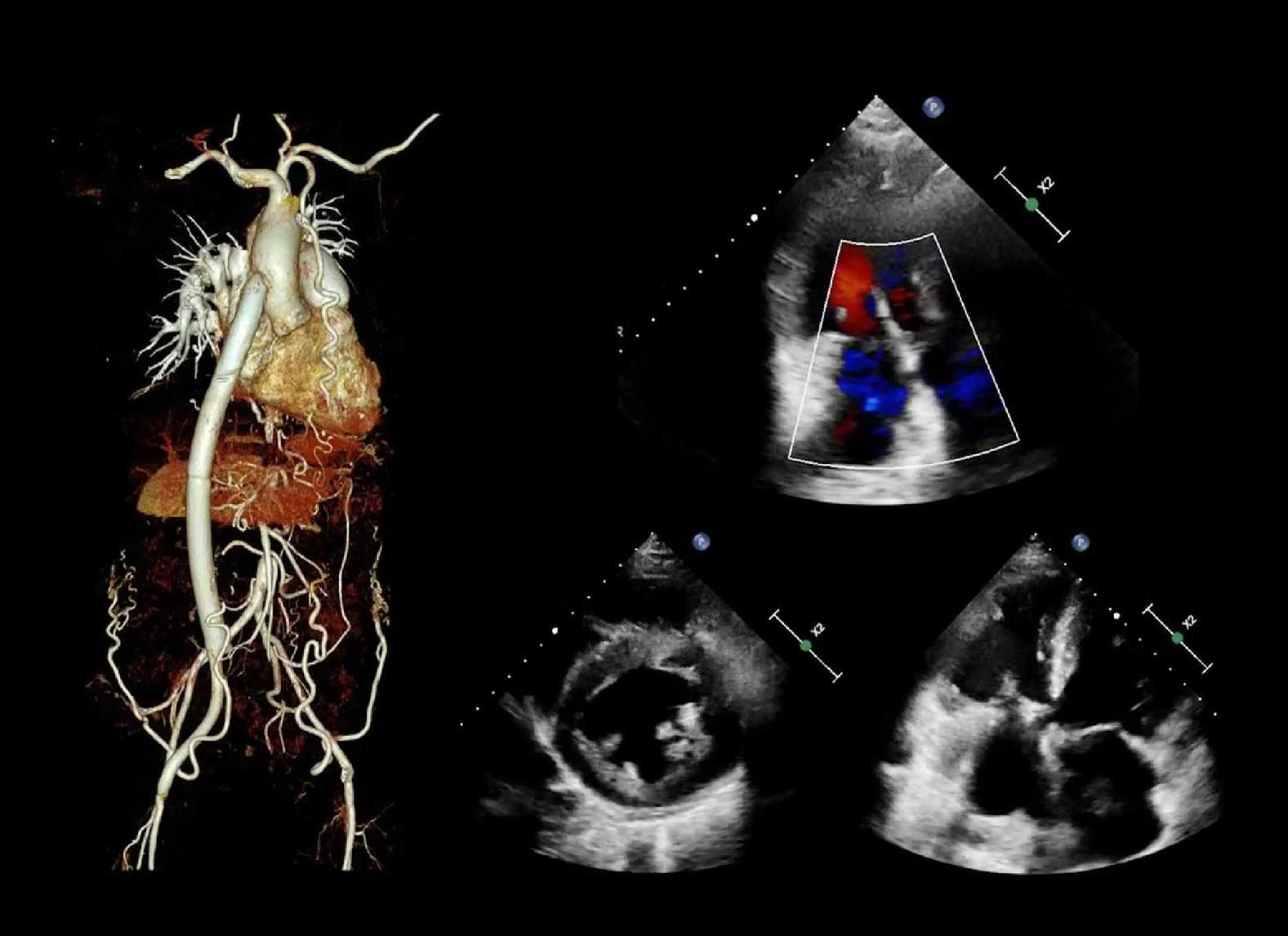

Supplement: Video 1 — Comprehensive visualization of mini-access ascending aorto-bifemoral bypass with tricuspid annuloplasty in this patient with mid-aortic syndrome and severe tricuspid regurgitation. Video available at: https://www.jtcvs.org/article/S2666-2507(25)00068-9/fulltext. [file fx2.jpg]
